# The predictive power of organizational identification on teachers' organizational citizenship behaviors

**DOI:** 10.3389/fpsyg.2026.1818062

**Published:** 2026-04-24

**Authors:** Hamza Öz, Erdal Meriç

**Affiliations:** 1 Yozgat Bozok University, Yozgat, Türkiye; 2 Ordu University, Ordu, Türkiye

**Keywords:** educational management, organizational citizenship behavior, organizational identification, school administration, social identity theory

## Abstract

This research aimed to reveal the relationship between teachers' perceptions of organizational identification and their organizational citizenship behaviors within the framework of social identity theory. This study, conducted using a correlational survey model from quantitative research methods, has organizational identification as its independent variable and the sub-dimensions of organizational citizenship behavior as its dependent variables. The research population consisted of teachers working in one province from each of Türkiye's seven geographical regions. The sample consisted of a total of 567 teachers selected using simple random sampling and who voluntarily participated in the study. The “Organizational Identification Scale” and the “Organizational Citizenship Behavior Scale” were used in the data collection process. In the analysis of the data, distribution characteristics were first examined, and it was determined that the data were suitable for parametric tests. Arithmetic mean and standard deviation values were calculated to describe the teachers' opinions; correlation and regression analyses were used to reveal the relationships between the variables. The findings showed that teachers' perceptions of organizational identification were at a high level. The study concluded that teachers' overall organizational citizenship behavior as well as their organizational citizenship behavior in the dimensions of sportsmanship and conscientiousness, were at a “very high” level, while their organizational citizenship behavior in the dimensions of altruism and civic virtue was at a “high” level. The research results revealed a moderate to high positive and significant correlation between organizational identification and organizational citizenship behavior. According to the regression analysis results, the perceived level of organizational identification among teachers is a significant determinant of organizational citizenship behavior. The results were interpreted within the framework of the relevant literature, and various suggestions for application and future research were developed in line with the findings.

## Introduction

Today's rapidly changing environmental conditions and increasing competition require organizations to achieve sustainable success not only by fulfilling the responsibilities outlined in their job descriptions, but also by exhibiting extra-role behaviors that voluntarily contribute to the organization's functioning. In this context, voluntary behaviors that significantly contribute to organizational efficiency and effectiveness are called organizational citizenship behaviors.

Many individual, managerial, and organizational factors influence the emergence of organizational citizenship behaviors. Identifying these factors helps organizations manage their human resources more effectively and maximize employee potential (Fan et al., 2023). In this context, organizational identification stands out as one of the determining factors in the development of these behaviors. Organizational identification refers to the process by which an individual integrates their values and goals with the goals and values of the institution they work for and sees themselves as an integral part of the organization. Individuals who develop a strong identification with their institution are thought to associate organizational success with their personal success and are more inclined to exhibit behaviors that contribute voluntarily beyond their job descriptions. Therefore, understanding the psychological dynamics underlying employee behavior is crucial-The development of human resources policies, the enhancement of organizational effectiveness, and the strengthening of employee-organization relations necessitate examining the relationship between organizational citizenship behavior and organizational identification. This is considered both a theoretical and practical need.

Organizational citizenship behaviors are among the important elements contributing to the strengthening of institutional effectiveness and leadership processes in schools (Shrestha and Subedi, 2020). In educational institutions, teachers are expected not only to fulfill their professional duties but also to demonstrate voluntary behaviors that contribute to the development of the school. Educational institutions, where social interactions are intense and shared values and goals are prioritized, are structures where social identity processes are strongly experienced. Teachers seeing themselves as valuable and respected members of their schools creates a foundation for them to take ownership of the school's successes and failures at a personal level. This sense of ownership is concretized through behaviors such as supporting colleagues, protecting the institution's image, undertaking voluntary responsibilities, and producing solutions to organizational problems.

Studies in the field of educational sciences provide an important theoretical foundation for understanding the effects of organizational citizenship behaviors on school effectiveness and teacher performance (Ng and Feldman, 2008; Tekin, 2018). Accordingly, examining the relationship between organizational citizenship behavior and organizational identification among teachers is crucial for improving school effectiveness and creating a sustainable collaborative culture in educational institutions. Social identity theory offers a strong theoretical framework for explaining this relationship. According to social identity theory, developed by Tajfel and Turner, individuals shape their self-perception not only through individual characteristics but also through the social groups to which they belong. As an individual's positive evaluation of their group increases, group membership becomes a central element of the self, supporting the emergence of behaviors that benefit the group. From this perspective, it is considered an expected outcome that individuals who establish a strong identity bond with their organization will exhibit voluntary behaviors that contribute to the organization's development.

From an organizational perspective, teachers are expected to exhibit organizational citizenship behaviors as a natural consequence of forming a strong identification with the schools where they work. Teachers' perceptions of the extent to which they are supported by the organization play a decisive role strengthening and the formation of organizational identification (Meriç et al., 2019). As the perceived level of organizational support increases, the emotional bonds individuals form with the organization strengthen, and their sense of belonging increases. According to the Organizational Support Theory developed by Robert Eisenberger and his colleagues, employees' perceptions of the extent to which they appreciate the organization's contributions and how much importance the organization places on their wellbeing determine the quality of their relationship with the organization (Eisenberger et al., 1986).

In educational institutions, teachers feeling supported by administrators and the institution contributes to their perception of the school not only as a workplace but also as a social structure to which they feel they belong. This strengthens teachers' organizational identification levels, creating a suitable psychological foundation for the development of organizational citizenship behaviors. This research is expected to make significant contributions to understanding how to encourage volunteerism and collaborative behaviors in schools by revealing the relationship between teachers' perceptions of organizational citizenship behaviors and their organizational identification. Furthermore, the study is anticipated to guide school administrators in developing strategies to increase teachers' commitment and identity levels.

In literature reveals that organizational identification and organizational citizenship behavior variables are mostly addressed under separate headings; however, there are also studies examining the relationship between these variables and different organizational outcomes (Çevik, 2025). Studies examining the relationships between organizational identification and variables such as organizational justice (Uzun, 2018a; Polat, 2022), organizational support and trust (Uzun, 2018b), burnout (Avanzi et al., 2015), and presenteeism (Öztürk Çiftci et al., 2018) exist in a teacher sample. On the other hand, the relationships between organizational citizenship behavior and concepts such as responsible leadership (Luo et al., 2025), self-efficacy (Choong and Ng, 2024), organizational commitment (Gökyer and Koçak, 2020), organizational trust (Gürbüz and Dede, 2017; Yilmaz, 2009), organizational health (Buluç, 2008), and organizational culture (Ipek, 2012) have also been investigated, and it has been emphasized that these behaviors are influenced by many individual and organizational factors (Erdogan and Bauer, 2014; Lee et al., 2019). However, it is observed that the number of studies directly examining the relationship between organizational identification and organizational citizenship behavior is limited (Demir, 2015; Edwards and Peccei, 2010; Luo et al., 2025). This situation strengthens the potential of the current research to fill the gap in the relevant literature and to offer both theoretical and practical contributions.

A review of the literature reveals that organizational citizenship behaviors are mostly examined through variables such as organizational justice, leadership, and organizational support; while identification, although a fundamental explanatory element, is examined in a relatively limited way. In this context, perceived organizational support offers a complementary mechanism explaining the development of identification, while Social Identity Theory forms the basic theoretical framework explaining how identification transforms into organizational citizenship behaviors.

Organizational citizenship behaviors are considered a critical element for the long-term success of organizations (Savaş, 2025). It is thought that, in addition to individual and organizational factors, teachers' level of organizational identification can also be influential in the emergence of these behaviors. Accordingly, examining the relationship between teachers' organizational identification levels and organizational citizenship behaviors from the perspective of social identity theory is considered important in terms of filling the gap in the literature. In this respect, it can be stated that the research has the potential to produce meaningful results, both theoretically and practically, in the fields of organizational behavior and educational management.

In recent years, the processes of teacher commitment, belonging, and identity development within educational organizations have increasingly become a subject of research. It is known that the psychological bond teachers form with their institutions is related to many outcome variables such as professional motivation, job satisfaction, school effectiveness, and organizational commitment. However, theoretical discussions explaining how the identity bond teachers develop with their schools translates into voluntary out-of-role behaviors are limited. In particular, the question of how organizational identification influences organizational citizenship behaviors such as supporting colleagues, voluntarily contributing to school development, and assuming institutional responsibility, through which psychological mechanisms, needs further explanation in the literature. In this regard, Social Identity Theory offers a powerful theoretical framework explaining how the identity bond individuals form with their groups encourages group-benefiting behaviors. Therefore, examining the relationship between teachers' organizational identity identification and organizational citizenship behaviors within this theoretical perspective will contribute to a better understanding of the psychological processes explaining teachers' voluntary contributions to their schools. In this respect, the present research aims to make a theoretical contribution to the literature on teacher belonging and organizational behavior by revealing how the identity bond that teachers establish with their organizations is reflected in voluntary behaviors within the school context.

## Theoretical framework

### Organizational citizenship behavior

Organizational Citizenship Behavior (OCB) is defined as a set of behaviors that go beyond simply fulfilling tasks and technical competencies, supporting the organizational, social, and psychological environment and facilitating the execution of tasks (Borman, 2004). However, OCB is also described as employees voluntarily contributing to the functioning of the organization beyond their formal job descriptions and supporting the achievement of organizational goals beyond their role boundaries (Dipaola and Tschannen-Moran, 2001; Organ, 2018).

In the literature, organizational citizenship behavior is addressed in five basic dimensions: altruism (helpfulness/cooperation), courtesy, sportsmanship, conscientiousness, and civic virtue (Organ, 1989). The altruism dimension encompasses behaviors such as employees supporting newcomers, assisting colleagues with heavy workloads, sharing information and materials related to work processes, and providing guidance on equipment use (Podsakoff et al., 2009, 2000). Courtesy refers to establishing respectful and constructive communication among organization members (Deluga, 1995; Nuesca and Balacy, 2019).

The sportsmanship dimension is defined as employees avoiding negative attitudes and behaviors toward the organization and maintaining a positive outlook despite problems encountered within the organization (Somech and Ron, 2007). Conscientiousness refers to employees acting responsibly, safeguarding the organization's interests, and performing their duties with high diligence (Barksdale and Werner, 2001). Civic virtue is defined as employees actively participating in organizational management, attaching importance to meetings and events, showing interest in organizational policies, and prioritizing the interests of the institution (Jurewicz, 2004). Furthermore, Kim and Park (2019) emphasize that the trust that develops between the organization and its employees has positive effects on civic virtue.

### Organizational identification

The concept of organizational identification is defined as a fundamental construct that helps understand the relationship an individual establishes with the organization (Tompkins and Cheney, 1985). Mael and Ashforth (1992) explain organizational identification as an individual developing a sense of belonging to a particular organization and perceiving the organization's successes or failures as part of their own experience. Similarly, Dutton et al. (1994) consider organizational identification as the cognitive link established between an individual's self-definition and the organization's identification.

Organizational identification is not limited solely to employees psychologically seeing themselves as an integral part of the organization (Scott and Lane, 2000); organizational identification is also considered a dynamic process in which individual goals and organizational goals become increasingly aligned (Ashforth and Mael, 1989). Within this framework, organizational identification is viewed as a multidimensional construct reflecting the extent to which employees establish strong relationships with the organization, demonstrate solidarity in difficult circumstances, develop positive attitudes and behaviors toward the organization, and integrate themselves with the organization's identification (Mael and Ashforth, 1992).

This concept, frequently used in organizational structures within the context of social identity theory, is said to become more pronounced when employees feel they share a common destiny with the organization (Abrams et al., 1998; Barattucci et al., 2021). This perspective demonstrates that organizational identification forms an important psychological basis for shaping employees' attitudes and behaviors toward the organization.

### Social identity theory

Social identity theory posits that individuals' self-perceptions are influenced not only by their personal characteristics but also by the social groups to which they belong (Tajfel and Turner, 2004). This theoretical approach provides an important framework for understanding social groups formed within organizations and the interactions between these groups. Individuals develop social identities through the relationships they establish with the groups they are a part of. Since organizations are structures where strong social bonds are formed for individuals, the relationships that employees develop with their organizations can be evaluated within the scope of the social identity process.

Social identity theory suggests that individuals utilize three basic mental processes when classifying themselves and others: social categorization, social identification, and social comparison. Social categorization refers to the process by which individuals position themselves and others within specific groups in order to make sense of their social environment. Social identification is concerned with an individual seeing themselves as a member of a group and making this membership a part of their self. Social comparison involves individuals comparing the groups they belong to with other groups to evaluate the value, status, and prestige of the group (Yaşar, 2022).

It is stated that teachers with a strong sense of organizational identity embrace the aims and values of the school, consider the success of the institution as part of their personal responsibility, and that this encourages a high level of organizational citizenship behavior (Organ et al., 2005; Podsakoff et al., 2000). This shows that organizational identification has a significant and positive effect on organizational citizenship behaviors (Riketta, 2005).

### Organizational support theory

Organizational support is considered an important psychological antecedent in the development of organizational identity (Eisenberger et al., 1986). Perceived organizational support is an implicit psychological contract that regulates the reciprocal interaction between employees and the organization (Turnley et al., 2003). Eisenberger et al. (1986) define perceived organizational support as employees' perceptions of the extent to which the organization values their contributions and wellbeing. In this context, organizational support refers to situations where organizational values prioritize employee wellbeing and include practices aimed at improving their overall wellbeing.

Perceived organizational support reflects employees' perceptions of the value the organization places on them and the extent to which their contributions are appreciated. At the heart of this concept lies the organization's attentive, caring, and supportive attitude toward its employees (Kim and Park, 2019). According to organizational support theory, employees develop more positive attitudes toward the organization and show stronger motivation to improve their performance and contribute to the organization when they feel supported and valued by the organization (Rhoades and Eisenberger, 2002). In this context, perceived organizational support is considered an important factor that strengthens employees' identification with the organization, paving the way for the emergence of organizational citizenship behaviors.

### Linking organizational identification and organizational citizenship behavior

Integrating social identity theory and organizational support theory provides a comprehensive framework for understanding the relationship between organizational identification and organizational citizenship behavior. Social identity theory suggests that individuals who strongly identify with a group are more likely to engage in behaviors that benefit that group. In organizational contexts, this implies that employees who identify with their organization will be more willing to contribute to organizational goals through voluntary actions.

Organizational citizenship behaviors can therefore be interpreted as behavioral manifestations of organizational identification. Teachers who perceive themselves as integral members of their schools may feel responsible for the wellbeing and success of the institution, which may motivate them to support colleagues, participate in institutional activities, and contribute to school improvement initiatives. Moreover, perceptions of organizational support may strengthen teachers' identification with their schools, thereby indirectly fostering organizational citizenship behaviors. From this perspective, organizational identification represents a key psychological mechanism through which teachers' voluntary contributions to their schools can be understood. Accordingly, examining the relationship between teachers' organizational identification and their organizational citizenship behaviors provides an important opportunity to better understand the psychological processes underlying voluntary and cooperative behaviors in educational organizations.

## Methodology

### Purpose of research

This research was conducted within the framework of social identity theory to examine the extent to which the organizational identification levels of teachers working in public schools predict their organizational citizenship behaviors. In line with this general objective, the following questions were addressed in the research:

1. What are the perception levels of teachers regarding their organizational identification and organizational citizenship behaviors?
2. What is the relationship between teachers' perceptions of organizational identification and their organizational citizenship behaviors?
3. To what extent do teachers' perceptions of organizational identification predict organizational citizenship behavior and its sub-dimensions (altruism, sportsmanship, conscientiousness, and civic virtue)?

### Research model

This research, which examines the relationship between teachers' organizational identification and organizational citizenship behaviors, was designed in accordance with the correlational survey model of quantitative research methodology. Research using the correlational survey model aims to determine the effect, relationship, and degree of this relationship of variables causing a phenomenon or event (Fraenkel and Wallen, 2009; Karasar, 2016).

### Study group

The study population consisted of teachers working in one city from each of Türkiye's seven different regions. The sample comprised 567 teachers selected from the population using simple random sampling and who voluntarily agreed to participate in the study. Analysis revealed that 258 (45.5%) of the participants were female and 309 (54.5%) were male; 454 (80.1%) were married and 113 (19.9%) were single. In terms of subject area, 192 (33.9%) were classroom teachers and 375 (66.1%) were subject teachers. Regarding educational background, 455 (80.2%) held a bachelor's degree and 112 (19.8%) held a master's degree. According to educational level, 207 (36.5%) were primary school teachers, 169 (29.8%) were middle school teachers, and 191 (33.7%) were high school teachers. Of the participants, 142 (25%) have 1-10 years, 165 (29.1%) have 11-20 years, and 260 (45.9%) have 21 years or more of professional experience. Regarding their length of service at their current schools, 253 (44.6%) have 1-5 years, 182 (32.1%) have 6-10 years, and 132 (23.3%) have 11 years or more of service.

### Data collection tools

The “Organizational Citizenship Behavior Scale,” developed by Podsakoff and MacKenzie (1989) and adapted into Turkish by Polat (2007), and the “Organizational Identification Scale,” developed by Mael and Ashforth (1992) and adapted into Turkish by Tak and Aydemir (2004), were used as data collection instruments in this study.

### Organizational Citizenship Behavior Scale

The “Organizational Citizenship Behavior Scale,” developed by Podsakoff and MacKenzie (1989) and adapted into Turkish by Polat (2007), initially consisted of five dimensions, but Polat (2007) study restructured it into four dimensions. Comprising a total of 20 items and four sub-dimensions, the scale includes eight items in the altruism dimension, four items in the sportsmanship dimension (these items will be reverse-coded), four items in the conscientiousness dimension, and four items in the civic virtue dimension. The scale items, prepared in a five-point Likert format, range from “Never” (1) to “Always” (5). Higher scores on each item indicate a higher perception in that dimension. Confirmatory factor analysis (CFA) was conducted to evaluate the construct validity of the Organizational Citizenship Behavior scale. It was determined that item loading values ranged from 0.45 to 0.82, the four-dimensional structure of the scale was preserved, and the model generally showed excellent and good fit levels (X^2^/sd = 2.05, CFI = 0.95, NFI = 0.91, GFI = 0.94, AGFI = 0.93, TLI = 0.94, IFI = 0.95, RMSEA = 0.04, SRMR = 0.04, RMR = 0.02). Items from the “altruism” and “kindness” dimensions of the original scale were grouped into a single dimension and used as the “altruism” dimension. In this study, the internal consistency coefficients of the scale were found to be Cronbach's Alpha 0.86 for the overall scale and 0.81, 0.70, 0.71, and 0.77 for the sub-dimensions, respectively.

### Organizational Identification Scale

The “Organizational Identification Scale,” developed by Mael and Ashforth (1992) and adapted into Turkish by Tak and Aydemir (2004), is a one-dimensional scale consisting of six items. The scale items, prepared in a five-point Likert format, are scored between “Strongly disagree” (1) and “Strongly agree” (5). Higher scores obtained from each item of the scale indicate a higher perception of organizational identification. Confirmatory Factor Analysis (CFA) was conducted to evaluate the construct validity of the Organizational Identification Scale. It was determined that item loading values ranged from 0.50 to 0.74, the unidimensional structure of the scale was preserved, and the model generally showed an excellent level of fit (X^2^/sd = 1.70, CFI = 0.99, NFI = 0.99, GFI = 0.99, AGFI = 0.98, TLI = 0.99, IFI = 0.99, RMSEA = 0.04, SRMR = 0.02, RMR = 0.02). In this study, the internal consistency coefficients of the scale were found to be Cronbach's Alpha 0.78 for the overall scale.

### Data collection and analysis

Research data were collected through an online survey created by transferring personal information forms and scale items to Google Forms. The research process consisted of several stages. First, ethical approval was obtained from the Ordu University Educational Research Ethics Committee (decision no. 2025/114 dated June 24, 2025). The study was conducted in accordance with the principles of the Helsinki Declaration, which emphasizes voluntary participation and ensures the anonymity of the data collection process. Participants were informed that they could withdraw from the study at any time, and informed consent was obtained before data collection. The collected data were analyzed using the SPSS statistical software package, and the analysis procedure was carried out in three stages.

In the first stage, the dataset was examined for missing, erroneous, and outlier values, as well as multicollinearity. In the second stage, normality assumptions were examined, and in the third stage, analyses addressing the research questions were performed. No missing data was identified. The survey forms completed by 600 teachers who voluntarily participated in the study were transferred to the SPSS program. To identify univariate outliers, z-scores exceeding ±3 were considered extreme values; Accordingly, 33 cases were identified as outliers and removed from the dataset. Multivariate outliers were then assessed using Mahalanobis distance, and no cases with values below 0.001 were detected. Cook distance was also calculated to examine influential observations in more detail, and no value exceeded 1.00. Consequently, cases identified as extreme were carefully reviewed, and those defined as anomalous observations were removed from the dataset to minimize their disproportionate impact on statistical estimates and to enhance the robustness and reliability of the findings. Analyses were then performed with the remaining 567 valid cases. Multicollinearity assumptions were tested by examining variance inflation factor (VIF) values, all of which were below 5, indicating no multicollinearity concern (Topal et al., 2010).

In the second stage, the distributional characteristics of the data were evaluated. Descriptive statistics regarding organizational citizenship behavior and organizational identification were examined to determine normality. For the total organizational identification score, skewness was calculated as−0.212 and kurtosis as 0.213. Skewness values for the total organizational citizenship behavior score and its sub-dimensions ranged from−0.890 to 0.121, while kurtosis values ranged from−0.684 to 0.213. According to George and Mallery (2010), skewness and kurtosis values within the ±1 range indicate a normal distribution. The normality of the data was also evaluated using Q-Q and histogram plots. The analysis revealed that the values observed in the Q-Q plots were approximately aligned with the reference line; and the histogram plots showed a bell-shaped and symmetrical distribution. These findings demonstrated that the variables met the normality assumptions. Therefore, parametric statistical tests were used for intergroup comparisons (Tabachnick and Fidell, 2013).

In the third stage, analyses addressing the research sub-questions were conducted. Initially, arithmetic mean scores were calculated for each dimension of the scales, and subsequent analyses were based on these mean values. Score ranges were interpreted as follows: 1.00-1.79 (very low), 1.80-2.59 (low), 2.60-3.39 (medium), 3.40-4.19 (high), and 4.20-5.00 (very high). These ranges were determined using a grouped scoring formula with absolute cutoff points. Relationships between variables were examined using the Pearson correlation coefficient (r). In addition, simple linear regression analysis was performed to determine the predictive power of independent variable on dependent variables. Standardized beta (β) coefficients and t-test results were considered when interpreting regression findings. All statistical analyses were performed at a significance level of 0.05%.

## Results

The findings section first presents the arithmetic mean and standard deviation values for teachers' organizational identification levels and organizational citizenship behaviors, then provides findings on the relationship between organizational identification and organizational citizenship behavior, and finally presents the results of the simple linear regression analysis conducted to determine the extent to which organizational identification predicts organizational citizenship behavior.

### Findings regarding teachers' organizational identification levels and organizational citizenship behaviors

In accordance with the first sub-objective of the research, the arithmetic mean and standard deviation values for teachers' organizational identification levels and organizational citizenship behaviors are shown in Table 1.

**Table 1:** Teachers' organizational identity levels and organizational citizenship behaviors.

Variables	*N*	*X*	SD
Organizational identification	567	3.49	0.67
Organizational citizenship behavior	567	4.22	0.38
*Altruism*	567	4.10	0.50
*Sportsmanship*	567	4.42	0.58
*Conscientiousness*	567	4.46	0.37
*Civic Virtue*	567	4.03	0.60

Table 1 shows that the arithmetic mean of teachers' organizational identification levels is *X* = 3.49 and the standard deviation is SD = 0.67. These findings indicate that teachers have a “high” level of organizational identification. The arithmetic mean of teachers' overall organizational citizenship behavior is *X* = 4.22 and the standard deviation is SD = 0.38. The arithmetic mean and standard deviation values of the sub-dimensions of organizational citizenship behavior are as follows: altruism dimension: *X* = 4.10, SD = 0.50; sportsmanship dimension: *X* = 4.42, SD = 0.58; conscientiousness dimension: *X* = 4.46, SD = 0.37; and civic virtue dimension: *X* = 4.03, SD = 0.60. These averages show that teachers' overall organizational citizenship behaviors, as well as their organizational citizenship behaviors in the dimensions of sportsmanship and conscientiousness, are at a “very high” level, while their organizational citizenship behaviors in the dimensions of altruism and civic virtue are at a “high” level.

### Findings regarding the relationship between organizational identity and organizational citizenship behavior

In accordance with the second sub-objective of the research, the results of the correlation analysis conducted on the relationship between organizational identification and organizational citizenship behavior are shown in Table 2.

**Table 2:** Relationships between organizational identification and organizational citizenship behavior.

Variables	(1)	(2)	(2.1)	(2.2)	(2.3)	(2.4)
Organizational identification	1	0.459^**^	0.376^**^	0.256^**^	0.190^**^	0.456^**^
Organizational citizenship behavior	0.459^**^	1	0.859^**^	0.601^**^	0.600^**^	0.775^**^
*Altruism*	0.376^**^	0.859^**^	1	0.267^**^	0.384^**^	0.552^**^
*Sportsmanship*	0.256^**^	0.601^**^	0.267^**^	1	0.286^**^	0.312^**^
*Conscientiousness*	0.190^**^	0.600^**^	0.384^**^	0.286^**^	1	0.357^**^
*Civic Virtue*	0.456^**^	0.775^**^	0.552^**^	0.312^**^	0.357^**^	1

^**^p < 0.01.

Table 2 shows a moderately positive and significant relationship between organizational identification and organizational citizenship behavior (*R* = 0.459, *p* < 0.001). This confirms the existence of the relationship, which is the main problem of the research.

### Findings regarding the predictive level of organizational identification on organizational citizenship behavior

In accordance with the third sub-objective of the research, the results of the simple linear regression analysis conducted to determine the extent to which organizational identification predicts organizational citizenship behavior and its sub-dimensions are shown in the tables.

Table 3 shows that organizational identification is a significant predictor of organizational citizenship behavior [*R* = 0.459, *F*_(1 − 565)_ = 150.490, *p* < 0.001]. The data in the table show that 21% (*R*^2^ = 0.210) of teachers' organizational citizenship behaviors are related to their organizational identification levels. In other words, it can be said that 21% of the total variance related to organizational citizenship behavior is explained by organizational identification. According to the calculated regression coefficient, a one-unit increase in organizational identification level causes a 0.257-unit increase in organizational citizenship behavior.

**Table 3:** Results of the simple linear regression analysis regarding the prediction of organizational citizenship behavior by organizational identity.

Predictor variable	Organizational citizenship behavior				
	*B*	SHB	β	*T*	*P*
Constant	3.322	0.075		44.525	0.000
Organizational identification	0.257	0.021	0.459	12.267	0.000

*R* = 0.459; *R*^2^ = 0.210; *F*_(1 − 565)_ = 150.490; *p* < 0.001.

Table 4 shows that organizational identification is a significant predictor of altruistic behavior [*R* = 0.376, *F*_(1 − 565)_ = 93.006, *p* < 0.001]. The data in the table show that 14% of teachers' altruistic behaviors (*R*^2^ = 0.141) are related to their organizational identification levels. In other words, it can be said that 14% of the total variance related to altruistic behavior is explained by organizational identification. According to the calculated regression coefficient, a one-unit increase in organizational identification level causes a 0.277-unit increase in altruistic behavior.

**Table 4:** Results of simple linear regression analysis on the prediction of altruistic behavior by organizational identity.

Predictor variable	Altruism				
	*B*	SHB	β	*T*	*P*
Constant	3.129	0.102		30.604	0.000
Organizational identification	0.277	0.029	0.376	9.644	0.000

*R* = 0.376; *R*^2^ = 0.141; *F*_(1 − 565)_ = 93.006; *p* < 0.001.

Table 5 shows that organizational identification is a significant predictor of gentlemanly behavior [*R* = 0.256, *F*_(1 − 565)_ = 39.609, *p* < 0.001]. The data in the table show that only 1% (*R*^2^ = 0.066) of the teachers' gentlemanly behavior is related to their organizational identification levels. In other words, it can be said that 1% of the total variance related to gentlemanly behavior is explained by organizational identification. According to the calculated regression coefficient, a one-unit increase in organizational identification level causes a 0.219-unit increase in gentlemanly behavior.

**Table 5:** Results of simple linear regression analysis on the prediction of sportsmanship behavior by organizational identity.

Predictor variable	Sportsmanship				
	*B*	SHB	β	*T*	*P*
Constant	3.652	0.124		29.471	0.000
Organizational Identification	0.219	0.035	0.256	6.294	0.000

*R* = 0.256; *R*^2^ = 0.066; *F*_(1 − 565)_ = 39.609; *p* < 0.001.

Table 6 shows that organizational identification is a significant predictor of conscientious behavior [*R* = 0.190, *F*_(1 − 565)_ = 21.140, *p* < 0.001]. The data in the table show that teachers' conscientious behaviors are only 0.04% dependent on their organizational identification levels (*R*^2^ = 0.036). In other words, it can be said that 0.04% of the total variance related to conscientious behavior is explained by organizational identification. According to the calculated regression coefficient, a one-unit increase in organizational identification level causes a 0.105-unit increase in conscientious behavior.

**Table 6:** Results of simple linear regression analysis on the prediction of conscientious behavior by organizational identification.

Predictor variable	Conscientiousness				
	*B*	SHB	β	*T*	*P*
Constant	4.096	0.081		50.637	0.000
Organizational identification	0.105	0.023	0.190	4.598	0.000

*R* = 0.190; *R*^2^ = 0.036; *F*_(1 − 565)_ = 21.140; *p* < 0.001.

When Table 7 is examined, it is seen that organizational identification is a significant determinant of civic virtue behavior [*R* = 0.456, *F*_(1 − 565)_ = 148.522, *p* < 0.001]. The data in the table show that 21% (*R*^2^ = 0.208) of the civic virtue behaviors of teachers are related to their organizational identification levels. In other words, it can be said that 21% of the total variance related to civic virtue behavior is explained by organizational identification. According to the calculated regression coefficient, a one-unit increase in organizational identification level causes a 0.408-unit increase in civic virtue behavior.

**Table 7:** Results of simple linear regression analysis on the prediction of civic virtue behavior by organizational identification.

Predictor variable	Civic virtue				
	*B*	SHB	β	*T*	*P*
Constant	2.605	0.119		21.855	0.000
Organizational identification	0.408	0.034	0.456	12.187	0.000

*R* = 0.456; *R*^2^ = 0.208; *F*_(1 − 565)_ = 148.522; *p* < 0.001.

## Discussion and conclusion

This research, conducted within the framework of social identity theory to examine the extent to which teachers' organizational identification levels predict their organizational citizenship behaviors, revealed that teachers' organizational identification levels are “high.” Many studies in the literature support this finding (Çavuşoglu, 2022; Hatipoglu, 2022; Polat, 2022; Uzun, 2018a,b). Teachers identifying with their schools by seeing themselves as part of the school and adopting its values and goals is crucial for the quality of the educational process. Teachers who identify with their schools view their roles not merely as an obligation, but as an integral part of the school's success. This leads to better preparation for their lessons and greater interaction with students.

The study concluded that teachers' overall organizational citizenship behaviors, as well as their behaviors in the dimensions of sportsmanship and conscientiousness, were at a “very high” level, while their behaviors in the dimensions of altruism and civic virtue were at a “high” level. Similarly, studies in the literature show that teachers' organizational citizenship behaviors are at high (Buluç, 2008; Gürbüz and Dede, 2017; Ipek, 2012; Shrestha and Subedi, 2020) and very high (Gökyer and Koçak, 2020) levels. This study found that teachers' overall organizational citizenship behaviors, and especially the dimensions of chivalry and conscientiousness, were at a “very high” level, indicating a strong sense of responsibility toward their schools and a willingness to exhibit voluntary behaviors that go beyond their job descriptions. High levels of cooperation and civic virtue behaviors suggest that teachers have positive tendencies toward supporting their colleagues and contributing to school development. However, the fact that these dimensions remain at a “high” rather than “very high” level suggests that teachers may be affected by factors such as workload, time constraints, bureaucratic processes, or limited opportunities for organizational participation. In particular, the relatively low levels of civic virtue behaviors suggest that teachers may face structural limitations in actively participating in school governance, voluntarily engaging in decision-making processes, or dedicating time to institutional development activities.

The study concluded that there is a moderately positive and significant relationship between organizational identification and organizational citizenship behavior. Similarly, positive relationships were found between organizational identification and the sub-dimensions of organizational citizenship behavior. This confirms the existence of the relationship, which is the main research problem. The results show that as teachers' organizational identities increase, their organizational citizenship behaviors also increase. This result is similar to the findings of many studies in the literature suggesting that teachers' positive perceptions of schools increase their organizational citizenship behaviors. Research in this context has found positive correlations between teachers' organizational identification levels (Demir, 2015), organizational trust (Gürbüz and Dede, 2017), organizational commitment (Gökyer and Koçak, 2020; Widodo et al., 2023), organizational socialization (Çavuşoglu, 2022), teacher empowerment (Bogler and Somech, 2004), and job satisfaction (Kartiko et al., 2023) and their organizational citizenship behaviors.

The study concluded that organizational identification is a significant predictor of organizational citizenship behavior, with 21% of teachers' organizational citizenship behaviors dependent on their level of organizational identification. The study also found that organizational identification significantly predicted all sub-dimensions of organizational citizenship behavior. Interpreted within the framework of social identity theory, this result can be interpreted as teachers identifying with their schools and perceiving the school as part of their own identity, thereby increasing voluntary behaviors that contribute to the functioning of the school but are not included in their formal job descriptions. The results demonstrate that organizational identification is a significant psychological mechanism for individuals to exhibit voluntary behaviors for the benefit of the organization, transcending their role descriptions. The findings are consistent with the core assumptions of social identity theory and support the decisive role of identity-based bonds that teachers form with their schools on behavioral outcomes.

According to social identity theory, individuals place the groups to which they belong at the center of their self-perception and evaluate the successes of these groups as their personal successes (Tajfel and Turner, 2004). In this context, organizational identification can be considered not only an attitude but also a powerful identity-based motivational source in the emergence of organizational citizenship behaviors. The findings are consistent with the core assumptions of social identity theory and support the decisive role of identity-based bonds that teachers establish with the school on behavioral outcomes. This result aligns with the findings of Ashforth and Mael (1989) and Riketta (2005); supporting the literature that organizational identity increases beneficial behaviors for the organization in the context of educational institutions.

According to organizational support theory, individuals establish stronger emotional bonds with the organization, and their sense of belonging is strengthened when they perceive the value the organization places on them (Eisenberger et al., 1986; Rhoades and Eisenberger, 2002). Although perception of organizational support was not directly tested as a variable in this study, organizational support theory offers a complementary theoretical framework explaining how organizational identification develops. In this context, teachers feeling supported by school administration and the institution can be considered an important prerequisite facilitating the development of organizational identification.

The findings of this study reveal a significant and positive relationship between teachers' levels of organizational identification and their organizational citizenship behaviors, supporting the explanatory power of social identity theory in the context of organizational behavior. According to social identity theory, individuals make the social groups to which they belong an important part of their self-concept and identify the success of these groups with their own success (Tajfel and Turner, 2004). In this context, the findings show that the identity-based bonds that teachers develop with their schools encourage them to exhibit voluntary behaviors that support the functioning of the organization. In other words, teachers' identification with their schools not only creates a psychological sense of belonging but also constitutes an important source of motivation that supports the emergence of extra-role behaviors for the benefit of the organization. These results are consistent with the findings of previous studies indicating that organizational identification increases individuals' voluntary contributions to organizational goals (Ashforth and Mael, 1989; Riketta, 2005).

The research findings also demonstrate that organizational identification is a significant determinant of organizational citizenship behavior, revealing that organizational identity functions as a fundamental psychological mechanism in the emergence of these behaviors. This is consistent with the “identity-based motivation” approach, which is increasingly emphasized in the organizational behavior literature. According to this approach, individuals tend to act in the best interests of the social groups to which they feel they belong by internalizing the values and goals of these groups (Ellemers et al., 2004; van Knippenberg, 2000). When evaluated within the context of educational organizations, teachers viewing their schools as part of their own identity can encourage them to engage in voluntary behaviors that contribute to the school's development, going beyond simply fulfilling their job descriptions. In this respect, the research findings contribute to the educational organizations literature by revealing that organizational identification is an important conceptual variable in explaining teachers' organizational citizenship behaviors.

This research also offers important implications from the perspective of organizational support theory. Organizational support theory suggests that employees' perceptions of how much an organization values them and cares about their wellbeing strengthen the psychological bonds they form with the organization (Eisenberger et al., 1986; Rhoades and Eisenberger, 2002). While perceived organizational support was not directly measured in this study, the high level of organizational identification development among teachers suggests that supportive organizational climates in educational institutions may facilitate identity formation. Therefore, this study points to the indirect role that perceived organizational support may play in the relationship between organizational identification and organizational citizenship behaviors, offering a new theoretical area of discussion for future research.

Finally, this research proposes a multi-layered model explaining teachers' organizational behavior by offering an integrative framework between social identity theory, the organizational identity approach, and organizational support theory. While social identity theory explains the behavioral consequences of identity-based bonds that individuals form with organizations (Tajfel and Turner, 2004), Organizational Support Theory sheds light on the organizational conditions under which these identity bonds can be strengthened (Rhoades and Eisenberger, 2002). Within this framework, the research findings reveal that the identity-based bonds teachers form with their schools are a significant determinant of organizational citizenship behaviors, contributing to theoretical discussions on understanding voluntary and cooperative behaviors in educational organizations.

### Practical implications for educational practice

The findings of this study provide several practical implications for school management and educational leadership. First, the results indicate that teachers' organizational identification is an important predictor of organizational citizenship behaviors. Therefore, school administrators should adopt leadership practices that strengthen teachers' sense of belonging and identification with their schools. Creating a supportive organizational climate, recognizing teachers' contributions, and encouraging participation in decision-making processes may help enhance teachers' identification with their institutions.

Second, school leaders may foster organizational citizenship behaviors by promoting collaborative cultures within schools. Opportunities for professional collaboration, peer support, and shared decision-making processes may encourage teachers to engage in voluntary behaviors that contribute to school improvement. Previous studies also suggest that supportive leadership practices and participatory management styles can strengthen teachers' organizational commitment and voluntary contributions to institutional goals (Bogler and Somech, 2023; Somech and Ron, 2007).

Finally, strengthening teachers' organizational identification may also contribute to improving school effectiveness. Teachers who perceive themselves as integral members of their schools are more likely to take responsibility for institutional success, support their colleagues, and actively participate in activities that enhance the overall functioning of the school. Therefore, policies aimed at strengthening teacher engagement and institutional belonging may play an important role in promoting positive organizational behaviors in educational settings.

### Limitations and directions for future research

Despite its contributions, this study has several limitations that should be acknowledged. First, the research employed a cross-sectional research design, which limits the ability to draw causal conclusions about the relationship between organizational identification and organizational citizenship behavior. Although the findings reveal significant associations between these variables, longitudinal or experimental research designs would provide stronger evidence regarding causal relationships.

Second, the study sample consisted only of public school teachers in Türkiye. Therefore, the generalizability of the findings may be limited to similar educational contexts. Future studies may examine the relationship between organizational identification and organizational citizenship behavior in different educational systems, private schools, or higher education institutions to determine whether similar patterns emerge.

Third, the study focused primarily on the relationship between organizational identification and organizational citizenship behavior. However, previous research suggests that various organizational and psychological factors—such as leadership styles, perceived organizational support, organizational justice, and school climate—may also influence teachers' voluntary behaviors (Podsakoff et al., 2000; Rhoades and Eisenberger, 2002). Future research may develop more comprehensive models by incorporating these variables as mediating or moderating factors.

## Data Availability

The original contributions presented in the study are included in the article/supplementary material, further inquiries can be directed to the corresponding author.
